# Insights into Endothelin Receptors in Pulmonary Hypertension

**DOI:** 10.3390/ijms241210206

**Published:** 2023-06-16

**Authors:** Ruiqi Liu, Tianyi Yuan, Ranran Wang, Difei Gong, Shoubao Wang, Guanhua Du, Lianhua Fang

**Affiliations:** 1 State Key Laboratory of Bioactive Substances and Functions of Natural Medicines, Institute of Materia Medica, Chinese Academy of Medical Sciences and Peking Union Medical College, Beijing 100050, China; liuruiqi@imm.ac.cn (R.L.); wangranran@imm.ac.cn (R.W.); gongdf@imm.ac.cn (D.G.); 2 Beijing Key Laboratory of Drug Targets Identification and Drug Screening, Institute of Materia Medica, Chinese Academy of Medical Sciences and Peking Union Medical College, Beijing 100050, China; yuantianyi@imm.ac.cn (T.Y.); shoubaowang@imm.ac.cn (S.W.)

**Keywords:** pulmonary hypertension, endothelin receptor, antagonist, drug target

## Abstract

Pulmonary hypertension (PH) is a disease which affects the cardiopulmonary system; it is defined as a mean pulmonary artery pressure (mPAP) > 20 mmHg as measured by right heart catheterization at rest, and is caused by complex and diverse mechanisms. In response to stimuli such as hypoxia and ischemia, the expression and synthesis of endothelin (ET) increase, leading to the activation of various signaling pathways downstream of it and producing effects such as the induction of abnormal vascular proliferation during the development of the disease. This paper reviews the regulation of endothelin receptors and their pathways in normal physiological processes and disease processes, and describes the mechanistic roles of ET receptor antagonists that are currently approved and used in clinical studies. Current clinical researches on ET are focused on the development of multi-target combinations and novel delivery methods to improve efficacy and patient compliance while reducing side effects. In this review, future research directions and trends of ET targets are described, including monotherapy and precision medicine.

## 1. Introduction

Pulmonary hypertension (PH) is a pathophysiological disorder in which the pressure in the pulmonary arteries rises due to increased pulmonary vascular resistance, most recently defined as a mean pulmonary artery pressure (mPAP) > 20 mmHg measured by right heart catheterization at rest at sea level (different altitudes can be converted to sea level). In order to distinguish PH with regard to different factors such as pulmonary vascular disease and left heart disease, the two indicators of pulmonary vascular resistance (PVR) and pulmonary arterial wedge pressure (PAWP) are usually introduced for definition. According to the 2022 ESC/ERS Guidelines, pre-capillary PH is hemodynamically defined as mPAP > 20 mmHg, PAWP ≤ 15 mmHg, and PVR > 2 Wood units (WU), while the post-capillary differences are PAWP > 15 mmHg and PVR ≤ 2 WU [1,2].

PH can lead to progressive dyspnea, fatigue, chest pain, syncope, oedema, palpitations, and eventually right heart failure [3]. This disease has complex mechanisms and diverse etiologies, and is currently classified into five main groups based on different pathological features: Group I—pulmonary arterial hypertension; Group II—PH due to heart disease; Group III—PH due to hypoxia and lung disease; Group IV—chronic thromboembolic PH; and Group V—PH due to other causes [4]. Based on PVR and PAWP levels, another classification method is to define patients with PH as pre-capillary PH, isolated post-capillary PH (IpcPH), or combined pre/post-capillary PH (CpcPH) [5]. Among these, PH associated with left heart disease is mainly linked with the two categories of IpcPH and CpcPH, while the rest of the categories belong to pre-capillary PH [1,5].

The etiology and pathogenesis of PH are diverse and complex, and are associated with many individual and environmental factors, usually leading to pulmonary vasoconstriction, the abnormal proliferation of pulmonary vascular smooth muscle, and pulmonary vascular remodeling [3]. Endothelin receptor antagonists (ERAs) are a powerful class of vasodilators and antiproliferative agents, and are currently commonly targeted for the treatment of PH. From the perspective of pathogenesis, in addition to targeting endothelin (ET) receptors to cause vasodilation, there are research advances being made in the fields of cell proliferation and anti-apoptosis, gene mutation, epigenetic dysregulation, metabolism, and immunity [6].

In addition to environmental factors (e.g., hypoxia, air pollution) and endothelial dysfunction, mutations in bone morphogenetic protein receptor 2 (BMPR2), activator receptor-like kinase 1 (ALK1), endoglin, and other genetic predispositions [7] are multiple factors contributing to PH, as are somatic circulatory factors such as pro-coagulation and inflammation and the expressions of many ion channels and receptors [8].

However, targeting endothelial function remains an important avenue for treatment, and this is currently the first choice for PH treatment. Combinations targeting the ET receptor pathway, the development of new formulations, and the expansion of indications remain advanced and are promising areas of research. Therefore, in this review we focus on the pathological changes in ET and its signaling pathway in the progression of PH as well as the drugs and latest clinical studies employed in achieving this target.

## 2. Background and Mechanism of PH

### 2.1. Pathological Process of PH

PH is a disease of the cardiopulmonary unit that affects pulmonary circulation and the right ventricle (RV). Although PH is divided into five major categories based on different and somewhat heterogeneous etiologies, in general their pathogeneses have many similarities. They are all characterized by the excessive proliferation of vascular cells, the increased deposition of the extracellular matrix, and the accumulation of inflammatory cells within the pulmonary vascular wall, which together lead to increased PVR [9]. In addition, other underlying causes such as congenital heart disease, systemic connective tissue disease, chronic lung disease, and left heart disease are potential factors that promote pulmonary vascular disease [10].

**Figure 1:**
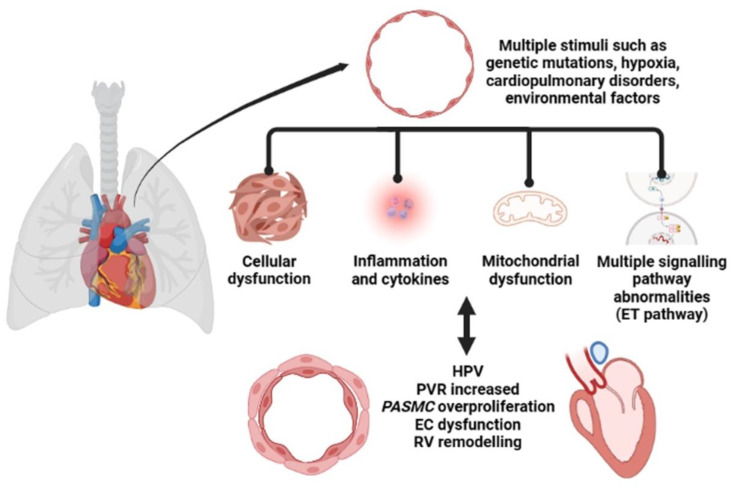
The pathological process of pulmonary hypertension (PH).

The pathological features of PH include endothelial cell (EC) dysfunction, abnormal signaling, and the abnormal proliferation of multiple cells and blood vessels, leading to the progressive evolution of arteriolar intimal proliferation with centripetal or eccentric laminar sclerosis, medial hypertrophy, and adventitial proliferation. It is also accompanied by variable inflammatory response and occasional fibrin-like necrosis [11], as shown in Figure 1. Currently, in addition to the widely recognized abnormalities in cell proliferation and function, the bioenergetics aspects of cell-related metabolic pathways as well as mitochondrial abnormalities are becoming increasingly important in PH [12]. Hypoxemia results in the need for higher cardiac output to maintain adequate tissue oxygenation, which is more difficult to achieve with a higher afterload [13]. Under normal conditions, the RV wall is thin compared to the left ventricle (LV), and low loads are sufficient for pulmonary oxygen circulation. Under normal physiological conditions, an appropriate increase in pulmonary artery pressure (PAP) facilitates systemic oxygen circulation [14]. When pressure increases slowly, RV first maintains output by compensating for the increase in wall thickness. However, this ventricular remodeling is not infinite and eventually leads to right heart failure [15]. The increase in resistance due to pulmonary vascular remodeling, which may be fourfold or higher, is the most important indicator of hemodynamic changes in PH [16].

Among the many stimuli, hypoxemia plays an important role in the production of PH. Hypoxic pulmonary vasoconstriction (HPV) due to acute hypoxia can lead to acute PH, and consequently to high altitude pulmonary edema. In contrast, chronic hypoxic exposure induces pulmonary vascular remodeling and the development of sustained PH, which increases the RV pressure load and eventually leads to right heart failure or even death [17]. HPV is a homeostatic mechanism that optimizes oxygen uptake by matching lung perfusion to ventilation. Local HPV is not accompanied by an increase in PAP; however, in the presence of total alveolar hypoxia, HPV involves the entire pulmonary circulation, resulting in increased PVR and elevated PAP [18]. Mitochondrial sensors present within the pulmonary artery smooth muscle cell (PASMC) respond to changes in oxygen tension by altering the production of reactive oxygen species (ROS), which are then rapidly and locally converted to H_2_O_2_ through mitochondrial superoxide dismutase 2 (SOD2) and regulate the activity of ion channels such as potassium (K^+^) and calcium (Ca^2+^). The simultaneous increase in combined nitrogen species inactivates various enzymes and vasodilatory factors in the nitric oxide (NO) pathway and promotes pulmonary vasoconstriction [19].

When pulmonary vasoconstriction persists over a long period of time, it causes vascular occlusion and the sustained elevation of PVP. This pathological status leads to the recruitment of perivascular inflammatory cells and factors with chronic inflammation and autoimmune features. In turn, certain inflammatory factors exacerbate pulmonary vasoconstriction and are closely associated with proliferation and remodeling of pulmonary vascular, ultimately leading to the development and progression of PH.

### 2.2. Signaling Pathways in PH

In the process of pulmonary endothelial dysfunction, endothelium-dependent vasodilatation becomes impaired. Many signaling pathways are involved in this process, with metabolic changes, ROS production, and disordered processes of different chemokines, cytokines, and growth factors. This eventually leads to impaired angiogenesis and repair mechanisms, which plays a major role in pulmonary vascular remodeling [20]. Many of these signaling pathways are associated with ET.

The small guanosine triphosphate binding protein ras homolog gene family member A (Rho A) and its major effector molecules, Rho-related kinases (ROCKs), play important roles in the cardiovascular system and have been found to regulate a wide range of essential cellular functions such as contraction, motility, proliferation, and apoptosis [21]. The Rho/ROCKs signaling pathway is a common signal transduction pathway in body tissues. Rho/ROCK can be activated by a variety of upstream stimulus signals, including ET-1, NO, and angiotensin II (Ang II), as well as by oxidative stress [22]. One study demonstrated the increased expression of Rho A, ROCK1, and ROCK2 in PH lung tissue and RV tissue, suggesting the activation of the Rho A/ROCK pathway and confirming that this pathway is associated with vascular remodeling in PH [23].

Additionally, factors such as hypoxia-inducible factor-1 (HIF-1α), which has an important role in PH, can regulate ET production and EC migration [24]. After a period of hypoxemia, a number of hypoxia-sensitive inflammatory responses and proliferative pathways are activated, leading to increased vascular resistance and even pulmonary vascular remodeling [25,26]. The exposure of pulmonary artery ECs to hypoxia in humans can cause an increase in HIF-1 expression, and the heterozygous expression of HIF-1 and HIF-2 in mice can have a protective effect against PH [27,28]. The increased expression of HIF-1α during hypoxia leads to the increased expression of the closely related transforming growth factor-beta 1 (TGF-β1), fibroblast growth factors (FGFs), and vascular endothelial growth factor A (VEGFA) along with its receptor vascular endothelial growth factor receptor 2 (VEGFR2), among others.

On the other hand, the enhancement of TGF-β1 stimulates an increase in platelet-derived growth factor beta (PDGFβ), leading to over-proliferation of ECs by stimulating VEGFA expression, all of which has been demonstrated in the pulmonary artery vessels of patients [29]. Additionally, this can activate the Rho A/ROCK signaling pathway, thereby promoting the occurrence and development of PH [30]. This further includes the release of different chemokines, cytokines, and growth factors from the endothelium and the increased expression of adhesion molecules such as E-selectin, intercellular adhesion molecule 1, and vascular cell adhesion molecules [20].

In addition to the pathways highlighted above, the AMP-activated protein kinases (AMPK), Notch, extracellular-signal-regulated kinase (ERK), c-jun N-terminal kinase (JNK), and P38 mitogen-activated protein kinase (MAPK) signaling pathways, which are associated with cellular energy state and vascular morphogenesis, play important roles in vascular development and differentiation [31,32,33]. Similarly, upregulated TRB3 [34] and abnormal resistin-like molecule β gene expression [35] both have regulatory effects on the MAPK signaling pathway. The more studied pathways include the NO pathway, where established drugs including phosphodiesterase (PDE5) inhibitors block the breakdown of cGMP and soluble guanylate cyclase activators (sGCS) act synergistically with endogenous NO to directly stimulate sGC and raise cGMP, and the prostacyclin pathway, which increases cAMP, promotes vasodilation, and inhibits platelet aggregation and smooth muscle cell proliferation [36]. Representative drugs include prostacyclin analogues and prostacyclin receptor agonists. Additionally, there are multiple pathways related to hypoxia, stress, and energy metabolism.

Many signaling pathways play a role in determining disease progression during the development of PH, and a large number of them function as upstream or downstream molecules of the ET signaling pathway. This indicates the importance of ET and its pathways in PH; the potential regulatory role of modulating ET receptors for the disease is very broad, and a very worthy target for research. Therefore, we next elaborate on ET receptors and their pathways.

## 3. Endothelin Receptors and Their Pathway Mechanisms

### 3.1. Endothelin Subtypes and Distribution

ET was originally identified as a potent vasoconstrictor peptide from porcine aortic ECs, which consist of twenty-one amino acid residues with a hydrophobic C terminus linked by two sets of intra-chain disulfide bonds and having a vasopressor effect. Similar to many peptide hormones and neuropeptides, ET is produced in ECs from a pre-peptide of approximately two hundred amino acids [37]. Based on the deduced amino acid sequences, Akihiro Inoue et al. cloned three different human ET-related genes by screening a genomic DNA library with synthetic oligonucleotide probes encoding partial sequences of ET at low hybridization intensity: synthesized endothelin-1 (ET-1), endothelin-2 (ET-2), and endothelin-3 (ET-3). Their biological activity was tested by contraction assays in isolated porcine coronary artery strips and intravenous injection assays in anesthetized rats. The results showed that three isomers of human ET exist, differing in structure and activity, but all being antihypertensive in vivo and potent arterial smooth muscle constrictors in vitro. The vasoconstrictor activity in terms of the maximum tension induced was ET-2 > ET-1 > ET-3, an order that is correlated with the hydrophobicity of the peptide [38,39].

The ET family is widely distributed in a variety of organs and systems in the human body, although different isomers are mainly distributed in slightly different organs. Of the ET family, the mature ET-1 peptide is synthesized in almost all kinds of cells and is highly expressed in ECs [40]. In response to stimuli such as hypoxia, ischemia, and shear stress, the messenger RNA (mRNA) for ET-1 undergoes transcription and rapidly synthesizes ET-1 [41]. In contrast to ET-1, ET-2 is mainly distributed in the kidney and small intestine. It is involved in a variety of biological activities in different systems, such as alveolarization, intestinal contraction, thermoregulation, and ovulation [42,43]. ET-3 is found mainly in the brain, and may be involved in the functional regulation of many neurons and glial cells. In addition, it is distributed in the lungs, kidney, and gastrointestinal tract [44].

### 3.2. Classification of Endothelin Receptors

As ET is hydrophilic and cannot cross the plasma membrane, it must bind to a specific cell surface receptor to function. There are two main subtypes of ET receptors, namely, ET_A_ and ET_B_; they belong to the G protein-coupled receptor superfamily, which consists of approximately four hundred amino acids, including seven transmembrane structural domains containing 22–26 hydrophobic amino acids [45]. The ET_A_ receptor is ET isoform selective and binds ET-1 and ET-2 with higher affinity than ET-3, whereas ET_B_ has the same affinity for all three isoforms and is not isoform selective [46]. There are two different basic functions of ET_B_ receptors; one is located on ECs that activate NO release, the other on vascular smooth muscle cells that mediate vasoconstriction [47,48,49].

**Figure 2:**
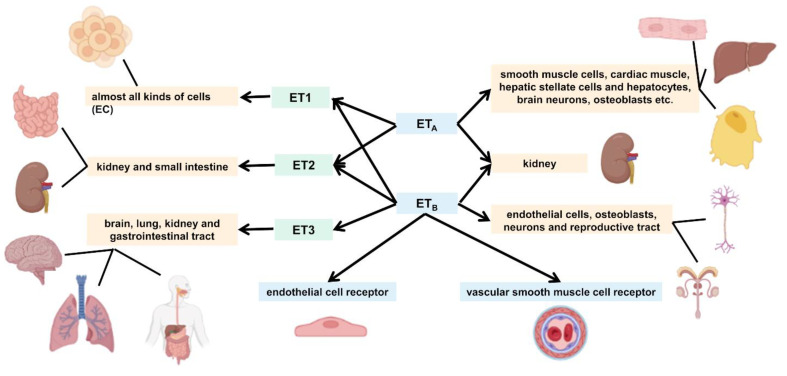
Endothelin (ET) isoforms and ET receptor subtypes.

In the human body, both receptors are abundantly distributed in the kidney, while ET_A_ receptors are found mainly in smooth muscle cells, cardiac muscle, hepatic stellate cells and hepatocytes, brain neurons, osteoblasts, etc. ET_B_ receptors are found mainly in ECs, osteoblasts, neurons of the central and peripheral nervous system, and various cells of the reproductive tract [50]. In the pulmonary arterial system, ET_A_ receptors mediate the proliferation of vascular smooth muscle cells and vasoconstriction, whereas ET_B_ receptors are located primarily on ECs, where they mediate vasodilation through the release of NO and prostaglandin-I-2 [51,52]. Figure 2 shows the relationship between the different isoforms of ET and the receptor subtypes, including their differences.

In addition, the ET_B_ receptor can initiate a positive autocrine loop that allows ET-1 to regulate the expression of its own genes and act as a “scavenger receptor” to remove circulating ET-1 via the lysosomal pathway [53,54]. The activated receptor couples to the effector system to produce second messengers such as inositol phosphate, diglycerides and calcium, ultimately producing the corresponding biological effects.

### 3.3. Endothelin Synthesis

**Figure 3:**
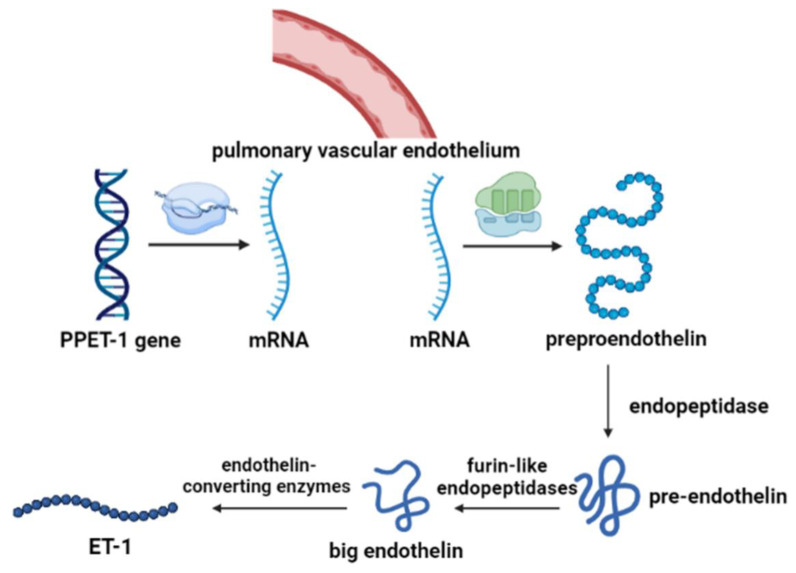
The synthesis process of ET-1.

ET is able to produce a variety of effects in lung tissue, including the proliferation of smooth muscle cells, pulmonary vascular contraction, secretion of mucus, stimulation of DNA synthesis, and changes in vascular permeability [55]. As shown in Figure 3, after being transcribed into mRNA by PPET gene regulation, ET is first translated into pre-proendothelin (PPET), which is regulated by c-fos and c-jun, nuclear factor-1, AP-1, and GATA-2. PPET is post-translationally cleaved at dibasic sites by furin-like endopeptidase to form proendothelin, then the biologically inactive big endothelin (big ETs), which in turn is cleaved at the Trp-Val for ET-1 (amino-terminal fragment) and ET-2 or at the Trp-Ile for ET-3 and a detectable carboxy-terminal fragment [56]. The formation of proendothelin activation receives regulation by the following pathways: the human PPET-1 gene is directly regulated by intracellular signaling mediated by protein kinase C via the trans-acting transcription factors FOS and JUN, and NF-1 binding elements mediate the induction of PPET-1 mRNA by TGF-β [57].

After synthesis, ET is secreted in two main ways. The first is through Weibel–Palade vesicles that act as storage sites for ET, fusing with the plasma membrane upon stimulation and releasing it via exocytosis. The other more dominant mechanism is the constitutive secretory pathway, which occurs in most endothelin-producing cell types. ET synthesized by vascular endothelial cells is released towards the basolateral side of the cell, and usually acts on smooth muscle cells. Therefore, tissue levels of ET are likely to be higher than plasma levels. ET is widely considered to be a paracrine/autocrine peptide rather than an “endocrine” peptide [58]. ET-1 can activate the PI3K/Akt pathway to stimulate endothelial NO synthase (eNOS) phosphorylation, leading to NO production, while NO can antagonize ET-1 by inhibiting preproET-1 transcription [59].

Because many ET-producing cells do not have storage vesicles or pathways to regulate secretion, the regulation of ET occurs primarily at the transcriptional stage [46,60]. At the molecular level, mRNA at the transcriptional level can be regulated to influence the synthesis of ET from ECs after intracellular processing. The synthesis of mRNA occurred in close proximity to the binding sites of ET in various tissues, and the binding sites of ET are evenly distributed in the lung, being distributed in decreasing order of frequency in the cardiovascular system, cardiac nerves, atria, ventricles, and coronary arteries. Of these, ET-1 and ET-3 are produced by the ECs of the large and small pulmonary vessels and the respiratory epithelium of the bronchiole, while ET-2 is mainly expressed by intestinal epithelial cells [60].

### 3.4. Regulation of the Endothelin Pathway

**Figure 4:**
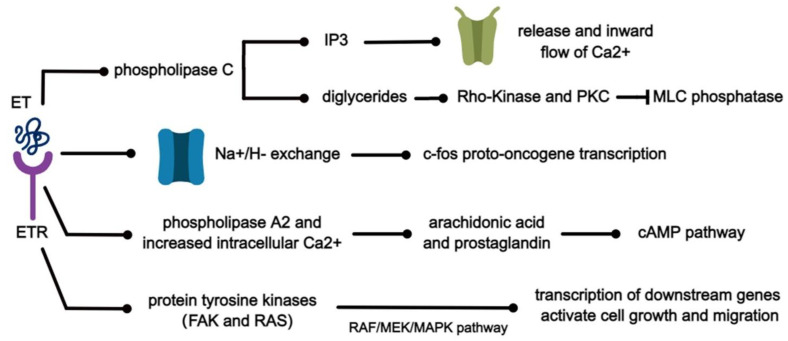
Signal pathways related to the ET pathway.

ET can interact with a variety of substances to initiate the corresponding signaling pathways and produce multiple biological effects. Several of these pathways are shown in Figure 4. When ET binds to its specific receptor, it activates phospholipase C and produces inositol triphosphate (IP3) and diglycerides [61]. It has been demonstrated in isolated rabbit arteries that IP3 subsequently diffuses to specific receptors on the endoplasmic reticulum, which in turn promote the release of Ca^2+^ from intracellular calcium stores and the inward flow of calcium through voltage-dependent calcium channels [62] and can induce mitogenesis. At the same time, the accumulation of diglycerides in turn activates Rho-Kinase and PKC, leading to the inhibition of myosin light chain (MLC) phosphatase [63]. Subsequently, the sensitivity of myofilaments to Ca^2+^ increases and the phosphorylation of MLC initiates the contraction of smooth muscle cells [62,64]. ET causes cytoplasmic alkalinization through the activation of Na^+^/H^−^ exchange, resulting in increased transcription of c-fos proto-oncogene and biochemical signals associated with proliferation [64]. In addition, ET-1 stimulates arachidonic acid production and prostaglandin release through the activation of phospholipase A2 and increased intracellular Ca^2+^, leading to the activation of the cAMP pathway. It can play an important role in lung diseases by mutually regulating protein expression through MAPK and AMPK signaling pathways, leading to the transcription of downstream genes and ultimately activating cell growth and migration [65,66].

ET can be affected by a wide range of substances in the body. Ang II can stimulate the release of immunoreactive ET from rat vascular smooth muscle cells via the AT1 receptor. Moreover, it shares a common receptor–effector transduction pathway with ET, and the two may promote vascular smooth muscle cell growth through a common intracellular signaling mechanism [67,68]. Its activity is potentiated by growth factors such as PDGF, epidermal growth factor (EGF), basic FGF, TGF-α, TGF-β, and insulin-like growth factor to synergistically stimulate DNA synthesis in vascular smooth muscle cells, fibroblasts, mesangial cells, melanocytes, osteoblasts, and ECs [60,69,70]. It has been shown that the full expression of ET-1 in vascular smooth muscle cells is potentiated by PDGF and EGF for pro-growth activity [71].

In ECs, TGF-β activates two different type I receptors, ALK5 and ALK1, through their respective ALK5/Smad2/3 and ALK1/Smad1/5 signaling pathways, with the former inhibiting and the latter stimulating EC proliferation and migration. TGF-β plays a key role in regulating the balance of ALK1 and ALK5 signaling in EC proliferation [72,73]. In studies targeting the TGF-b/ALK1/Endoglin signaling pathway, increased expression of ALK1 and ENG was found in the lung tissue and pulmonary ECs of patients with PH compared to the normal group. Increased TGF-β in the plasma and lungs led to the phosphorylation of Smad1/5/8, and induced pulmonary ECs to express ET-1, PDGFβ and FGF2. At the same time, despite reduced pulmonary vascular density, ENG-deficient mice were partially protected against chronic hypoxia-induced PH compared to wild-type mice [74].

## 4. Mechanism of the Endothelin Pathway and Its Antagonists in PH

### 4.1. The Role of Endothelin in the Disease Process

The importance of the ET pathway and its vital role in disease has been on the radar of researchers for three decades. In 1988, Masashi Yanagisawa and his team showed by in vitro and in vivo experiments with synthetic rat ET that it caused slow and sustained contraction of rat aortic strips and showed strong coronary constricting activity in isolated perfused rat hearts [75]. In humans, when high concentrations of ET-1 or ET-3 are provided via the forearm circulation, transient vasodilation occurs first, followed by sustained vasoconstriction, consistent with the effects on ET_B_ receptors. The systemic circulation shows a dose-dependent significant sustained increase in blood pressure that is largely dependent on renal, mesenteric and muscle vasoconstriction, whereas the pulmonary circulation is far less sensitive. ET is not only a vasoconstrictor; it appears to be associated with vascular and cardiac hypertrophy as well as with cardiovascular diseases such as arterial hypertension, PH, atherosclerosis, coronary artery disease, and cardiac and renal failure [76]. Overexpression of the ET-1 gene in blood vessels exacerbates vascular hypertrophy [77], though not remodeling.

In addition, ET induces cell differentiation by activating PKC to inhibit EGF-stimulated DNA synthesis, stimulates protein and RNA synthesis in smooth and cardiac muscle, and may play an important role in the development of cardiac diseases such as vascular hypertrophy and cardiac hypertrophy. For example, it acts as a mitogen and dedifferentiation factor in smooth muscle cells at the onset of atherosclerosis. Plasma ET-1 concentrations are higher in atherosclerotic patients than in normal subjects, which may be related to oxidative LDL stimulating ET-1 production in ECs [78].

Patients with PH have elevated levels of ET-1 in plasma and elevated ET-1 expression in pulmonary vascular ECs. Additionally, hypoxic or monocrotaline-induced rat models exhibited a lung-specific increase in ET-1 peptide, ET-1, and ET_A_ mRNA along with elevated PAP and RV hypertrophy. ET_A_/ET_B_ receptor antagonists have been shown to prevent and delay plasma ET-1 levels in the 2-week hypoxic prophylactic model only, though they have exhibited results of effectively improved RV hypertrophy and PAP [79,80]. In the systemic circulation, the ET_A_-selective antagonist BQ123 abolished the pressor effect of ET-1 in rats, while the ET_A_/ET_B_ antagonist PD145065 suppressed constriction of systemic vessels in response to ET. The impact of transcription factor signaling pathways on the disease process has been studied as well. In a Kruppel-like factor 4 knockout model (KLF4, a transcription factor expressed in the vascular endothelium) as well as at the protein level, it has been shown to have anti-inflammatory and antithrombotic effects [81,82] and to regulate the transcription of genes involved in three key pathways in the pathogenesis of PH, namely, the NO pathway, ET pathway and prostacyclin pathway [83].

### 4.2. ET_A_ Receptor Antagonists

ERAs have a long history of research and their mechanisms of action have been well studied. In studies of ERAs, the first to be tested in humans was a natural byproduct of *Streptomyces mistakii* fermentation, but was less potent in terms of binding and functional analysis. Selective ET_A_ receptor blockers reduced acute hypoxic PH in two models of hypoxia and group B streptococcal-induced acute PH in piglets, whereas they were not only ineffective but even exacerbated group B streptococcal-induced PH [84]. In acute hypoxia experiments with mature conscious horses, the ET_A_ receptor antagonist TBC11251 was found to not significantly affect the hemodynamic or ventilatory response to acute (10 min) hypoxia. In isolated equine pulmonary arteries, ET_A_ receptors mediated the contraction in response to ET-1, and TBC11251 effectively blocked ET-1-induced pulmonary vasoconstriction in horses, again demonstrating the role of ET in HPH at the animal level [85].

Similar results have been demonstrated in a variety of hypoxic and monocrotaline rat models, significantly reducing PH, RV hypertrophy, and RV remodeling [80], and in a post-reoxygenation neonatal piglet model showed decreased leukocyte-mediated injury and improved pulmonary function [86]. Additionally, in many studies decades ago, many antagonists showed similar protective effects on PH, such as the highly effective oral selective ET_A_ receptor antagonist TA-0201 [87]. Research continues on its broad effects and potential side effects to expand the indications and improve the efficacy of this target.

### 4.3. ET_B_ Receptor Antagonists

Similar results have been obtained when targeting ET_B_ receptors. It has been found that ET_B_ receptor deletion enhances the appearance of cellular and molecular markers associated with the pathobiology of PH and accelerates the disease progression, suggesting the regulation of the resting pulmonary vascular tone and an anti-proliferative protective role for ET_B_ receptors in pulmonary vascular homeostasis [88]. There have been many studies on many different targeting strategies over the years. As an antagonist targeting the effects of ET_B_ receptors on PH, Daniel S. Green’s team targeted receptor fragments via cell permeable peptides, which blocked ET_B_ receptor-mediated vasoconstriction on smooth muscle cells while maintaining the integrity of other functions. The peptide was found to impede the ET-1-promoted phosphorylation of ERK and Akt in pulmonary artery smooth muscle, significantly reducing RV systolic pressure and RV hypertrophy as well as significantly reducing fully muscularized vessels and alleviating the symptoms of PH [89].

However, studies in EC-specific ET_B_ knockout (KO) mice have shown that ET_B_-mediated NO/PGI2 release is reduced during hypoxia and that the concomitant absence of the NO/PGI2-mediated vasodilatory pathway may lead to disease progression, demonstrating the protective role of ET_B_ [90]. Moreover, the absence of ET_B_ signaling may further contribute to an increase in ET-1 [91].

## 5. Therapeutic Approaches

A variety of therapeutic modalities, such as exercise rehabilitation, oxygen therapy, anticoagulation, calcium channel blockers, diuretics, and anti-arrhythmic drugs, are currently used in clinical practice [92]. An imbalance between pulmonary vasodilator factors, including NO and PGI2, and vasoconstrictor factors such as ET-1, is considered to be an important factor in PH. Therefore, current therapeutic agents target the endothelial factors of vasodilation or constriction and proliferation.

The three main endothelial factor pathways currently used to target vasoconstriction and proliferation are the NO-cGMP signaling pathway (PDE5 inhibitors and soluble GC stimulators), ERAs, and the prostacyclin signaling pathway (prostacyclin analogs and IP receptor agonists) [13]. In addition to ERAs, drugs targeting other pathways can have a modulatory effect on ET. For example, both sildenafil and inhaled NO treatment can reduce HIF-1α and ET-1 levels in patients, demonstrating the broad role of ET targets [93]. The use of a single ERA or PDE5 inhibitor remains the initial treatment option for patients with PH [94].

### 5.1. Treatments Targeting Endothelin Receptors

Selective and non-selective ERAs are classical drug candidates currently being developed for potential clinical use in various diseases, including GMA-306 in PAH and BQ-123 in congestive heart failure, PD-161721 for atherosclerosis, enrasentan in myocardial infarction, sovateltide in cerebrovascular and coronary artery spasm, atrasentan in renal diseases, and more [45]. Researchers conducting a systematic review of randomized and semi-randomized trials that included patients with PH found that the ERA group increased exercise capacity, improved subjects’ WHO functional class, and produced favorable changes in cardiopulmonary hemodynamic variables. However, they were less effective in reducing dyspnoea and mortality. The combined use of ERAs and phosphodiesterase inhibitors may provide more benefit in PH [95].

Table 1 summarizes drugs in development that target ET receptors, covering those which are marketed, in clinical studies, in preclinical studies, and have been withdrawn, including their targets, doses, development institutions, and other basic information. The main drugs currently approved to target the ET pathway are bosentan, ambrisentan, and macitentan [96]. The role of oral ERAs in this disease was first demonstrated back in 1995, when the oral non-selective antagonist bosentan was found to have a preventive and reversal effect on pulmonary vasoconstriction and pulmonary vascular remodeling caused by acute and chronic hypoxia-induced PH [97]. Similar results were obtained in experiments with the orally administered selective ET_A_ receptor antagonist ZD1611 [98]. Most of the subsequent drug development was carried out following the ideas of bosentan-based structural modification and potency enhancement. Improvement and good tolerability of several secondary endpoints were evaluated in the Randomized Double-Blind Placebo-Controlled Multicenter Efficacy Study 1 and 2 (ARIES-1 and ARIES-2) randomized and controlled trials for the selective ET_A_ receptor antagonist ambrisentan [99,100], which has a lower risk of liver injury and less potential for drug interactions compared with bosentan [101]. Macitentan, also called Actelion-1 or ACT-064992, is a dual ET-1 receptor antagonist obtained by modifying the structure of bosentan [102,103]. The efficacy of macitentan in PH was investigated in the phase III SERAPHIN trial (NCT00660179), which showed a significant reduction in morbidity and mortality and an improvement in 6 min walk distance (6MWD) in patients with PAH. Its therapeutic effect was comparable to that of bosentan and aniracetam, and patients exhibited fewer adverse effects [104]. It had higher lipophilicity, increased receptor affinity, prolonged receptor binding, enhanced tissue penetration, and sustained antagonism of the ET-1 receptor [105]. In addition, it had activity on both ET_A_ and ET_B_ receptors, though it was much more selective for the ET_A_ receptor than the ET_B_ receptor in vitro.

However, there have been failures along the road of ERAs development as well. The selective ET_A_ receptor antagonist sitaxentan was withdrawn from the market in 2010 due to acute hepatotoxicity, and possible increased liver toxicity has been mentioned in systematic reviews [95,106]. In the Tsang JY trial, the non-selective ET receptor blocker tezosentan had minimal effect on post-acute pulmonary thromboembolism gas exchange, although it reduced the symptoms of PH in acute pulmonary thromboembolism-induced hypoxia PH [107]. Following the withdrawal of sitaxsentan, many ERA drug development projects have been aborted. PH and heart failure were the main indications in several clinical studies with tezosentan, and all were stopped by early 2010 for reasons including slow recruitment (NCT01094067, NCT01077297).

Currently, ambrisentan, bosentan, and macitentan have clinical applications and all improve symptoms and exercise capacity. Neither ambrisentan nor macitentan causes abnormal liver function such as the elevation of serum aminotransferase concentrations, though the former may cause peripheral edema and the latter may cause a decrease in hemoglobin in patients. Patients treated with bosentan should have monthly liver function tests, and it can produce many drug interactions [99,108,109].

### 5.2. Drugs under Clinical Investigation

In recent years, the combination of ERAs and PDE5 antagonists has become a hot topic in research and development to improve efficacy by targeting different pathways involved in disease pathogenesis. Studies of fixed-dose combinations (NCT03904693), combination formulations (CTR20211325), and tadalafil alone are all in clinical phase III. Two UK companies are conducting Phase 1 clinical trials of fixed-dose combinations of aniracetam and tadalafil (NCT02688387, JPRN-UMIN000005464).

Progress has been made in the study of a novel targeted drug, with preclinical studies by Zhang Cheng’s team on the ET_A_ receptor antagonist monoclonal antibody (MABs) getagozumab demonstrating its long-lasting and high effective action and good safety profile. It has been granted orphan drug designation by the FDA, and is expected to become a new therapeutic option [110]. 

Compared to small molecules, MABs are highly selective for target proteins, have fewer off-target, longer plasma half-lives, and improve patient compliance. They offer a wider range of therapeutic strategies, though they may cause immunogenic reactions in patients, potentially reducing their therapeutic efficacy. Between 2018 and 2020, Gmax Biopharm LLC in China conducted four clinical trials to validate the safety, tolerability, and pharmacokinetic profile of this monoclonal antibody (NCT04503733, CTR20200854, NCT04505137, ACTRN12618000121268), all of which are currently in the Phase I clinical stage. Another small molecule drug, SC-0062, is undergoing phase I trials in oral capsules (CTR20201868) conducted by the same Chinese company, and has been granted patents relating to pyrimidine sulfonamide derivatives and their preparation and medical applications. In addition to PH, the indications under investigation include immunological, endocrine, and metabolic diseases such as diabetic nephropathy, immunoglobulin A nephropathy, and high-altitude disease.

**Table 1:** Summary of clinical studies of endothelin receptor antagonists (ERA) drugs.

Drug	Dosage	Target	R&D Status	R&D Institutions	Country	Reference/Clinical Trial Identifier:
Macitentan	10 mg qd p.o.	Non-selective	Approved	Actelion Pharmaceuticals Ltd.	Switzerland	[111]
Ambrisentan	5 mg qd p.o. 10 mg qd p.o. if tolerated	ET_A_	Approved	Abbott Laboratories	United States	[112]
Bosentan	62.5 mg bid p.o. (4w) 125 mg bid p.o.	Non-selective	Approved	F. Hoffmann-La Roche Ltd.	Switzerland	[113]
Macitentan/Tadalafil	Macitentan 10 mg Tadalafil 20 mg	Non-selective PDE5A	Clinical Phase III	Actelion Pharmaceuticals Ltd.	Switzerland	NCT05236231
Ambrisentan/Tadalafil	Ambrisentan 10 mg Tadalafil 40 mg	ET_A_	Clinical Phase I	GSK Plc	United Kingdom	NCT02688387
Getagozumab	300–1800 mg i.v.	ET_A_	Clinical Phase I	Gmax Biopharm LLC	China	NCT04503733
Recombinant anti-human ET_A_ humanized monoclonal antibody	1500–2000 mg i.v.	ET_A_	Clinical Phase I	Gmax Biopharm LLC	China	NCT04505137
SC-0062	50 mg qd p.o.	ET_A_	Clinical Phase I	Shijiazhuang Zhikang Hongren New Drug Development Co Ltd.	China	CTR20201868
Sitaxentan Sodium	100 mg qd p.o.	ET_A_	Pre-clinical	Pfizer Inc.	United States	NCT01210443
Enrasentan	60–90 mg qd p.o.	Non-selective	Terminated	GSK Plc	United Kingdom	[114]
PD-156707	40 mg/kg qd p.o. for rats	ET_A_	Terminated	Pfizer Inc.	United States	[115]
Tezosentan disodium	5 mg/h i.v.	Non-selective	Terminated	F. Hoffmann-La Roche Ltd.	Switzerland	NCT01094067
ZD-1611	1–3 mg/kg qid p.o.	ET_A_	Terminated	AstraZeneca Pharmaceutical Co., Ltd.	China	[98]

Moreover, in Jin Cai’s work, a series of phenoxybutyric acid derivatives were designed and synthesized based on the characteristics of most non-peptide ERAs and it was demonstrated that one of these selective ET_A_ antagonists was effective in alleviating hypoxia PH and RV hypertrophy index, which may have potential for further development [116].

### 5.3. Problems and Prospects in Research and Development

Although current treatments targeting the ET receptor pathway have antiproliferative effects on PASMCs and delay the disease process, they do not reverse the progression of PH and have not been successful in improving survival. The current mortality rate of PH patients remains high, the prognosis remains unsatisfactory, and the situation may have become worse under the impact of the COVID-19 pandemic in the last three years. In recent years, research into targeting ET receptors using chemical entities other than small molecules, such as monoclonal antibody antagonists and selective peptide agonists and antagonists, has expanded rapidly, diversifying the targets in the ET signaling pathway. Monoclonal antibodies have low oral bioavailability due to their large molecular weight and poor membrane permeability; thus, oral bioavailability is sacrificed to extend therapy to other pathophysiological conditions. The emerging strategy is to target the dual vasoconstrictor targets angiotensin AT1 receptor and ET_A_ receptor and use the combination of two drugs with different targets (the ET_A_ antagonist aniracetam and the PDE5 inhibitor tadalafil) to improve the treatment of PH [117]. In addition, the induction of mild acidosis reverses PASMC phenotype conversion improves pulmonary hemodynamics and vascular function and attenuates pulmonary artery remodeling, which may be a complementary method for enhancing the effectiveness of vasodilators for PH [63].

More popular PH target research areas include hypoxia signaling pathways, cell metabolism, inflammation, cell proliferation, and personalized therapy. Beginning in 2010, the NHLBI has proposed the application of systems biology and histological thinking to develop models that better reflect disease states as a means of improving molecular-clinical phenotypic coupling of disease. Throughout the decade, significant progress has been made in these areas [118]. PH shares similar characteristics with cancer in terms of aberrant proliferation; therefore, a number of the approaches that have been applied to cancer treatment could be potential research directions for the treatment of PH, such as targeting transcription factors [119]. Altered epigenetic histone acetylation modifications are a widespread feature in PH, and by defining interaction networks and hierarchies, bioinformatics approaches can help to identify potentially successful transcription factor targets [120]. The application of histological ideas to the study of the PH lung transcriptome can provide a method for the identification of many pathways and the regulators within them [121]. Investigators have suggested that future therapeutic targets for PH could focus more on clinical endpoints of symptoms, quality of life and exercise capacity, surrogate endpoints, the understanding of treatment heterogeneity, and a review of responder analysis [122]. In addition, as PH is caused by abnormalities in multiple pathways in patients and our existing animal models are relatively homogeneous, it is difficult to simulate the clinical situations; this variability between animals and humans is one of the reasons why many drugs currently fail when they enter clinical studies.

In the future, personalized precision medicine targeting ET aspects may become a hot topic of research. By linking variations in patient genetics, i.e., DNA sequence variations that occur when individual nucleotides in the genome differ between individuals (single nucleotide polymorphisms, SNPs), drug targets such as disease-associated transmitter systems and drug treatment regimens can be tailored to individual patients. A key advance in this field is the experimental validation of genome-wide significant SNPs in five vascular diseases, with individuals carrying different levels of the minor allele having different levels of ET-1 and differing responsiveness to ET_A_ antagonism [123]. This study provides a theoretical basis for stratifying patients with regard to ET compound therapy by testing for SNPs, exemplifying the potential for the use of precision medicine in ET.

## 6. Conclusions

Due to multiple factors, PH is a complex disease of the cardiopulmonary vascular system that can lead to right heart failure and death. ET is a very important vasoconstrictor peptide that is widely distributed throughout the body and plays multiple roles in human physiological activities. An elevated expression of ET and elevated plasma levels acts through multiple signaling pathways to ultimately lead to elevated pulmonary vascular pressure, remodeling, and subsequent disease progression. In this review, we have discussed the pathological process of PH, focusing on the signaling pathways and mechanisms related to ET in disease development, and reviewed the synthesis, distribution, and effects of ET. In the clinical treatment of PH, ERAs are among the most common first-line drugs, and are pharmacologically effective in relieving symptoms. As a multi-causal and highly lethal disease, most current treatment options target symptoms and other related cardiopulmonary diseases while failing to reverse disease processes such as vascular remodeling.

ERAs have a relatively long history of development and use, and are a relatively mature treatment option in clinical practice. Clinical studies on ERAs are numerous, focusing on structural modifications of existing drugs, development of new targeted drugs, dosage forms, and multi-target combination studies with the aim of improving patient compliance and reducing side effects. With the rapid development of targeted immunotherapy in recent years, humanized monoclonal antibodies for ET receptors have made relatively good progress, resulting in a new turnaround of ET in the field of targeted therapy. As the development of genomics, transcriptomics, and other multidimensional genomics has progressed, researchers have discovered that suboptimal clinical efficacy may be related to individual differences, which in turn has led to a surge in research on personalized treatment for individual patients. In addition, with the development of bioinformatics such as system biology and network pharmacology, it is possible to obtain multi-target information on the disease and the target network relationship with ET. This is a very important non-experimental approach for target screening, multi-target combinations, and expansion of ET indications. For example, research on inflammatory pathways, metabolic disorders, and immune abnormalities has progressed in recent years, providing new potential targets for the development of new drugs for PH.

Overall, ET receptors play an important role in PH research as a classical target. Considering both efficacy and side effects, future studies on ERAs should always consider factors such as the patient’s age, gender, body mass index, ethnicity, and genetic susceptibility, as well as any comorbidities and their severity. Many new techniques and theoretical approaches that have been applied to other diseases can be considered as a reference for future research in this field. Moreover, the development of more clinically appropriate animal models is a key issue in improving the quality of drug development, and it is particularly important to review past studies in this regard and learn from them.

## Data Availability

No new data were created or analyzed in this study. Data sharing is not applicable to this article.
